# Beyond Quantum Physics

**DOI:** 10.4103/0973-6131.53836

**Published:** 2009

**Authors:** H R Nagendra

**Affiliations:** Swami Vivekananda Yoga Anusandhana Samsthana (A Yoga University), #19, Eknath Bhavan, Gavipuram, KG Nagar, Bangalore - 560019, India E-mail: hrn@vyasa.org

Modern Science has, over four centuries, made strides un-witnessed probably in the history of mankind. Scanning through the physical world with all its complications and complexities, the classical mechanics, relativistic mechanics, and quantum mechanics, we have understood the world around us with mathematical precision. At one time, a few decades back, we thought that matter and energy are two independent building bricks of our physical world.

However, we progressed to find the matter–energy continuum. Quarks as the packets of energy have shown us that everything in the physical world can be conceived as essential energy and is governed by the equation E= mc ^2^. As Fritj of Capra has put it, the science is in transition to move to understand the deeper and subtler dimensions of creation to bring into its fold consciousness. Attempts are continuously made to understand the facets of consciousness, spectrum of consciousness, etc. It is in this context, Prof Josephson tells often in his lectures that we need to go beyond quantum physics. He has great expectations in the ancient wisdom of the East in general and Upanishads in particular.

The 10 Upaniúads (Èùá, Kena, Kaûha, Muïãaaka, Mañdükya, Aitereya, Taittirèya, Chandogya, and Brhadaranyaka) forming the wisdom base for which India is known all over the world has the other dimensions of creation presented with such vividity that any scientist would get fascinated about it. The other specialty is that the techniques of Yoga provide the necessary skills to realize the truth or reality or pure consciousness by gaining mastery over the mind. Reality is at the base of all creation and this state of deepest silence of mind is also the supreme bliss, total knowledge, and the power unparalleled. The Taittèreya Upaniúat has promoted this wisdom base through it spaòca koúa viveka—the five layered existence of the whole creation of which the physical world is the grossest. Next to that are the subtle layers unseen by the eyes called práïamaya, Manomaya, and Vijòánamaya Koúás. The causal layer of consciousness is the ánandamaya koúa from where all creation emerges. A schematic of the same is self-explanatory. As a manifest of bliss, as waves in an ocean. The layer of bliss is a state where mind has gone to its deepest levels of silence with all pervasive expanses.

**Figure:**
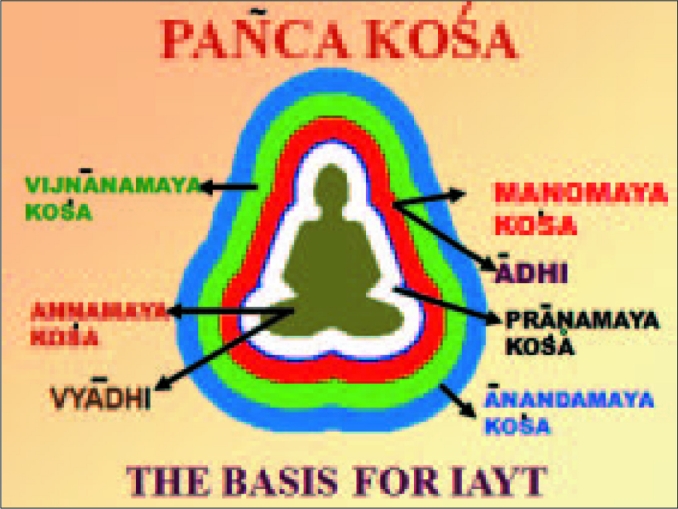
“Each soul is potentially divine. The goal of life is to manifest that Divinity within” – said Swami Vivekananda who enunciated the four streams of yoga for achieving this goal. IJOY is one such expression.

